# Direct Capture Methods Reveal Extensive Organohalide Chemical Space in Marine Environments

**DOI:** 10.3390/md24070237

**Published:** 2026-07-04

**Authors:** Alexander Bogdanov, Douglas Sweeney, Melissa L. Carter, Kayla Martin, Elena Beckhaus, Paul R. Jensen

**Affiliations:** Scripps Institution of Oceanography, University of California San Diego, La Jolla, CA 92093, USA

**Keywords:** SMIRC, marine natural products, metabolomics, novel compounds, halogenation

## Abstract

The vast majority of the ocean’s microbial natural product biosynthetic potential remains undescribed. To access this chemical diversity, we employed Small Molecule In Situ Resin Capture (SMIRC) across three ecologically distinct sites in San Diego, California. Using high-resolution LC-MS/MS, we detected spatial and temporal variability in the metabolomes captured. Low annotation rates and evidence of extensive halogenation supported the chemical novelty of the compounds captured. We detected rare chlorinated polyketides in the pinnaic acid class, previously known only from filter-feeding invertebrates. We also report the first detection of chlorosulfolipids in the Eastern Pacific Ocean including one that contained 11 chlorine atoms. We linked compound abundances to weekly phytoplankton counts to identify candidate producers and found evidence that different taxa produce chlorosulfolipids of different carbon chain lengths. This study provides evidence of the chemical novelty that can be captured directly from the environment and a framework for integrating environmental metabolomics with phytoplankton counts as a method to identify candidate compound producers.

## 1. Introduction

Microbial natural products remain one of our most important sources of chemical diversity [1]. Yet, the vast majority of Earth’s microbial natural product biosynthetic potential remains unrealized [2]. This is particularly true for marine environments, where it is estimated that <0.1% of bacteria have been obtained in culture [3] and thus are unavailable for traditional natural product discovery efforts. While new cultivation techniques [4], the use of elicitors [5], co-cultivation [6,7], and synthetic biology [8] have been used to access novel chemistry, the “dark chemical space” that compromises the majority of the ocean metabolome remains poorly explored for natural product discovery.

Recent advances in environmental metabolomics are providing new insights into the chemical diversity that can be detected in the marine environment [9]. Furthering these efforts are public data sharing platforms (https://massive.ucsd.edu, accessed on 2 July 2026) and analytical tools such as the GNPS ecosystem [10] and MASST [11], which provide rapid methods to identify compounds based on MS/MS data matching and determine compound distributions in nature, respectively. A distinct feature of marine natural products is the frequent incorporation of halogen atoms. [12,13]. Halogenation can increase biological activity [14], thus making these compounds of particular interest for drug discovery programs [15,16]. Both chlorine and bromine, which are abundant in seawater, possess isotopic ratios that make halogenated natural products readily detectable in mass spectrometry data. This feature provides a ‘chemical hook’ by which to prioritize halogenated natural products for identification.

We recently introduced a new approach to environmental metabolomics that we called Small Molecule In Situ Resin Capture (SMIRC) [17]. SMIRC was designed to extend environmental metabolomics to the discovery of new natural product scaffolds. Here we report on the chemical diversity captured using SMIRC at three locations around San Diego. We show evidence of chemical novelty and spatiotemporal variation in the metabolomes captured and describe efforts to link compounds to their microbiological origins based on phytoplankton counts.

## 2. Results

### 2.1. SMIRC Deployments

**Figure 1:**
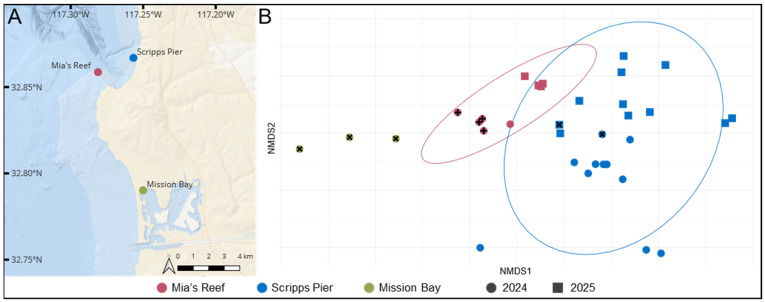
SMIRC deployment sites and metabolome similarities. (**A**) San Diego deployment locations (colored circles). (**B**) NMDS plot of metabolome Bray–Curtis dissimilarities (stress: 0.155) colored by site. Shape defines deployment year. Within shapes: × indicates used HP-20 resin, + indicates HP-20 agar, no symbol indicates new HP-20 resin. Plot created using combined positive and negative-mode LCMS data. Circles indicate 95% confidence intervals.

SMIRC was deployed at three locations around San Diego: Mia’s reef (32°51′31.0″ N 117°16′52.4″ W, −16–17 m depth, abbreviation MR), Mission Bay (32°47′24.7″ N 117°15′00.3″ W, −2 m depth, abbreviation MB), and the Scripps Pier seawater supply channel (32°52′00.8″ N 117°15′24.4″ W, abbreviation SP) (Figure 1A). The SP deployments were in some cases timed to coincide with visible phytoplankton blooms. In total, 34 individual resin packages were recovered from the three sites between June 2024 and September 2025 (22 SP, 9 MR and 3 MB; Supplementary Table S1). Across all sites, extract yields averaged 1.63 ± 0.81 mg/g (0.61–3.87 mg/g resin) for new HP-20 resin (*n* = 25). In five cases, we re-used HP-20, which resulted in lower yields of 0.75 ± 0.52 mg/g resin. In four cases (MR site only), the resin was embedded in agar to stimulate bacterial growth. This resulted in average yields of 2.13 ± 0.42 mg/g resin. When considering the deployment of new resin at SP, longer deployment times generated higher extract yields: <24 h average = 1.24 ± 0.32 mg/g (*n* = 8); 48 h average = 1.46 ± 0.21 mg/g (*n* = 5); 96 h average = 2.76 ± 0.64 mg/g (*n* = 6).

### 2.2. Spatiotemporal Metabolome Variability

**Figure 2:**
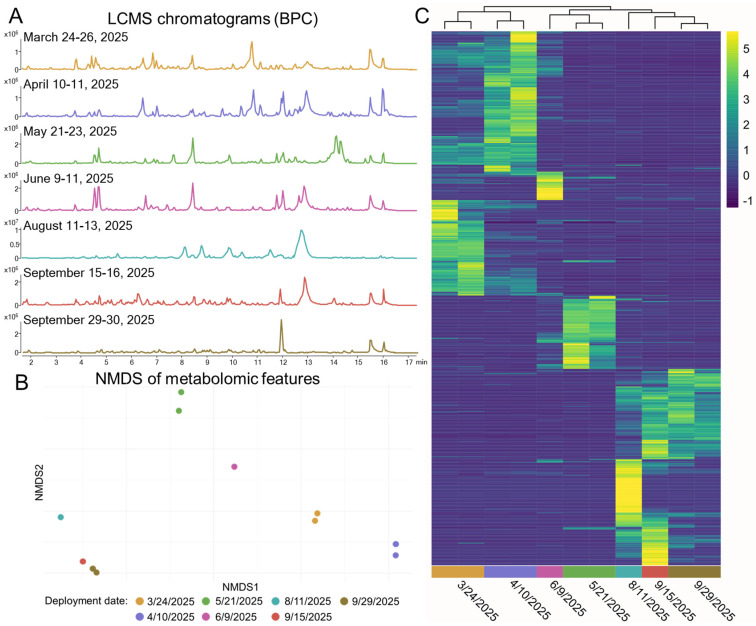
Temporal variability in Scripps Pier 2025 SMIRC metabolomes. (**A**) LCMS base peak chromatograms (BPC) (positive mode) from the seven 2025 SP deployments (only one replicate shown) reveal metabolomic changes over time. (**B**) NMDS plot of metabolome Bray–Curtis dissimilarities (stress: 0.043) reveals temporal variation. (**C**) Heatmap and hierarchical clustering (1000 most significant features, positive mode) reveal features that drive temporal variation.

LC-HR-ESIMS analysis of the 34 SMIRC extracts yielded 3843 and 3520 metabolite features (i.e., *m*/*z* values at a given retention time) in positive and negative mode, respectively. An NMDS plot of the combined features provided evidence that the metabolomes could be distinguished based on location (Figure 1B). While the deployment of used resin may contribute to the separation of the MB site, not all used resin samples grouped together suggesting that location is a stronger driver of metabolomic differences. Similarly, MR extracts separated from other sites regardless of resin type while the SP extracts obtained using new resin showed clear evidence of temporal separation. Manual inspection of the seven SP deployments made in 2025, all generated using new resin, supports changes in extract composition over time (Figure 2A). Further support for temporal separation comes from an NMDS plot of the 2025 SP metabolomes (Figure 2B). A heat map and hierarchical clustering analysis grouped replicate extracts together (when available) and revealed highly abundant features that were driving the differences among metabolomes (Figure 2C). Notably, extracts from temporally similar deployments clustered together in both the NMDS and hierarchical analyses. These results provide evidence that chemically distinct metabolomes can be captured from the same site over time thereby expanding the chemical diversity that can be obtained from a single location.

### 2.3. Compound Identification

Compounds were putatively identified based on MS/MS matches with spectra in the GNPS library [10]. Spectral matches were found for 115 of the 3843 positive-mode features (2.99%) and 32 of the 3520 negative-mode features (0.9%). The lower annotation rate for the negative-mode features is likely a reflection of the smaller number of negative-mode library spectra (~1,570,000 in positive mode vs. ~290,000 in negative mode as of March 2026). The low annotation rates highlight the potential chemical novelty of the SMIRC extracts. In the positive mode, 22 unique, high-confidence matches were detected (mass difference < 0.04 Da, number of shared fragment peaks > 8 or cosine score > 0.8, and manual verification; Supplementary Table S2, Supplementary Figure S1). The majority of these were to various lipids and conjugated cholic acids. Additionally, we detected cabrillostatin, which we characterized as part of a prior study [17], in all extracts. The negative-mode matches included polyphenolic compounds (diosmetin, rosmarinic acid, 3-(3-hydroxyphenyl)propionic acid), the fungal metabolite pyrenophorol, phosphatidylcholine, phosphatidylethanolamine and phosphatidylinositol lipids, sulfoursodeoxycholic acid, and the synthetic detergent decylbenzenesulfonic acid (Supplementary Table S3, Supplementary Figure S2). We also identified yessotoxin (*m*/*z* 1141.471 [M − H]^−^) at very low intensities in five SP extracts based on literature comparison [18].

The match with the highest number of shared peaks (*n* = 29) was to the cyclic lipopeptide surfactin C1 (Supplementary Figure S1), a membranolytic surfactant known from numerous microbial sources including a marine-derived *Bacillus* sp. [18,19]. Another confident match was to the mycosporine-like amino acid (MAA) usujirene (Supplementary Figure S1) or its stereoisomer palythene [20], which are indistinguishable by MS/MS. MAAs were first discovered in terrestrial fungi and later found in cyanobacteria, dinoflagellates, cnidarians, and mollusks [21]. They provide UV photoprotection and were detected at the highest levels from the shallow (<1 m) Mission Bay site and in one Scripps Pier extract obtained during a dinoflagellate bloom. Several lipid matches were to diacylglyceryl carboxyhydroxymethylcholines (DGCCs). After close inspection of the library entries and LCMS/MS data, the molecules bore only one fatty acid chain and thus were identified as monoacylglyceryl carboxyhydroxymethylcholines (MGCCs) or lyso-DGCCs. These polar betaine lipids have been reported from algae, diatoms, and dinoflagellates [22,23,24]. The MGCCs formed a distinct cluster in a molecular network (Supplementary Figure S3), with a majority annotated based on GNPS library entries or literature values [22]. Elevated levels of MGCC lipids were observed during dinoflagellate blooms (Supplementary Figure S4).

### 2.4. Halogenated Natural Products

Based on manual inspection of the LCMS data, we identified the monochlorinated cyanobacterial compounds malyngamide K (*m*/*z* 424.2424 [M + H]^+^) and malyngamide F or deoxy-epoxymalyngamide C (*m*/*z* 440.2532 [M + H]^+^) from the Mission Bay samples (Supplementary Figure S5). Of note, no match was found with the malyngamide K and F MS/MS spectra in the GNPS library, likely due to the use of different MS instruments. To date, malyngamides have only been reported from tropical *Moorea* (formerly *Lyngbya*) collections [25,26,27]. Their detection in Mission Bay compliments our 2024 report of apratoxin A from this same location [17]. Like the malyngamides, apratoxin A has only been reported from tropical *Moorea* collections [28]. Determining the biological origins of the compounds detected in temperate Mission Bay samples remains important given the impacts of *Moorea majuscula* toxins on human health [29].

**Figure 3:**
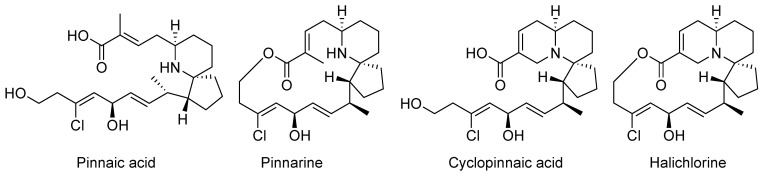
Halogenated compounds in the 6-aza-spiro[4.5]decane family. Pinnaic acid, pinnarine, and the proposed new compound cyclopinnaic acid were captured with SMIRC as part of this study and putatively identified based on HR-MS/MS data with absolute configurations inferred based on published structures.

From a single Scripps Pier extract (SP24-R2, June 2024), we detected 19 halogenated compounds in positive ionization mode (Table 1). Accurate mass and predicted molecular formula queries of the Dictionary of Natural Products (DNP) database revealed only two matches to the known natural products pinnaic acid [30] and pinnarine [31]. These structurally related chlorinated polyketides, together with tauropinnaic acid [30] and halichlorine [32], are the only members of a rare class of marine compounds containing a 6-aza-spiro[4.5]decane moiety (Figure 3). While the GNPS library does not contain ESI-MS/MS spectra for pinnaic acid or its derivatives, we could detect *m*/*z* 408 [M-H_2_O + H]^+^ and *m*/*z* 308 (6-aza-spiro[4.5]decane core) fragments in our MS/MS spectra that correspond to published reports [30], albeit at varying intensities likely due to different ionization techniques (ESI vs. EI-MS). After careful analysis, we verified the *m*/*z* 426.2375 [M + H]^+^ and *m*/*z* 408.2266 [M + H]^+^ ions and annotated most MS/MS peaks to plausible pinnaic acid and pinnarine fragments, respectively (Supplementary Figures S6 and S7). We also observed a closely related but apparently new compound with an *m*/*z* 424.2217 [M + H]^+^ and putatively assigned its structure as cyclopinnaic acid (Supplementary Figure S8).

All previously known pinnaic acid analogs were isolated from the bivalve *Pinna muricata* or the marine sponge *Halichondria okadai* [30,31,32]. Given their low yields, taxonomic distributions, and the filter-feeding lifestyles of the source organisms, they are likely of microbial origin. The recent report of pinnaic acid in SPATT extracts from Nigerian coastal waters [33] provides further support for this hypothesis. Interestingly, we consistently detected pinnaic acid or its derivatives from all three sites hinting towards a widespread, waterborne producer. The highest titers were observed in the Scripps Pier extracts and were not always correlated with visual plankton blooms (Supplementary Figure S9).

**Figure 4:**
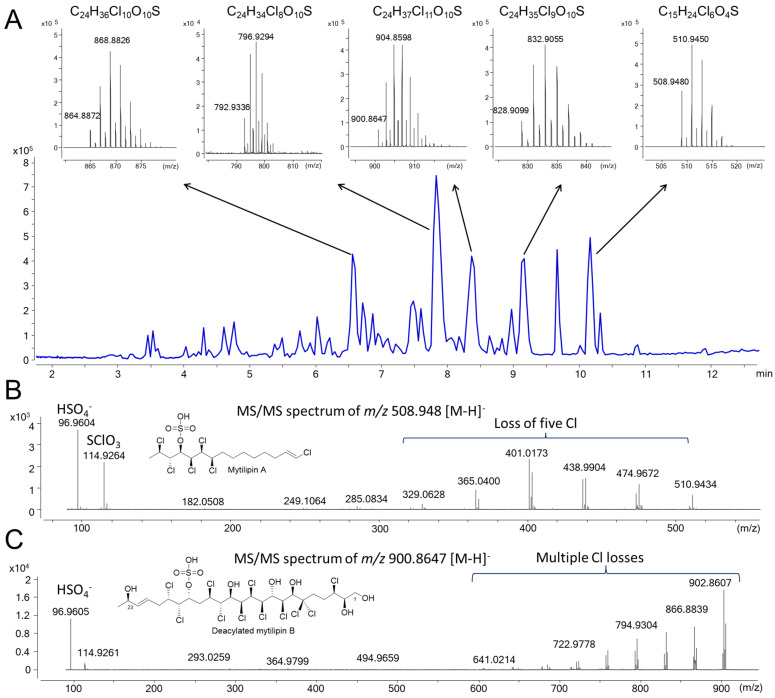
MS analysis of chlorosulfolipids captured by SMIRC. (**A**) Base peak chromatogram and MS spectra of major C_15_ and C_24_ chlorosulfolipids identified in the Scripps Pier extract generated from sample SP25-R5 collected in May 2025 following April–May 2025 *Pseudo-nitzschia* and *Alexandrium* blooms. (**B**) MS/MS spectrum of C_15_ chlorosulfolipid *m*/*z* 508.9480 [M − H]^−^ (calcd. for C_15_H_23_Cl_6_O_4_S 508.9454 [M − H]^−^, −5.1 ppm) putatively identified as mytilipin A. (**C**) MS/MS spectrum of C_24_ chlorosulfolipid *m*/*z* 900.8647 [M − H]^−^ (calcd. for C_24_H_36_Cl_11_O_10_S 900.8608 [M − H]^−^, 4.3 ppm) putatively identified as C-23 deacylated mytilipin B.

Additional compounds with isotopic patterns indicating halogenation were detected in the Scripps Pier SMIRC extracts generated after an upwelling event in April 2025. Interestingly, these compounds were only detected in negative mode and required optimizing the chromatographic conditions to improve peak shape and separation (see methods). Due to their complex isotopic patterns (Figure 4A), we used the “Isotope Distribution Calculator and Mass Spec Plotter” (https://www.sisweb.com/mstools/isotope.htm, accessed on 2 December 2025) to propose molecular formulas with up to 11 chlorine atoms per molecule. We queried the DNP and found mass matches to chlorosulfolipids. These compounds, which bear up to six chlorines, were first described from the fresh water chrysophyte (golden algae) *Ochromonas danica* [34] in the 1960s and later from *Poterioochromonas malhamensis* [35]. Following cases of shellfish poisonings in the Adriatic Sea, chlorosulfolipids with up to 11 chlorines were isolated from the mussel *Mytilus galloprovincialis* and reported as a suspected cause for diarrheic shellfish poisoning [36,37,38]. Based on de novo analysis of MS/MS spectra and comparison with literature reports, we putatively identified the structures of the chlorosulfolipids mytilipin A (Figure 4B) [37] (*m*/*z* 508.948 [M − H]^−^) and a new mytilipin B analog that lacks the C_16_ fatty acyl group at position C-23 (*m*/*z* 900.8647 [M − H]^−^, Figure 4C) [38]. MS/MS spectra of both compounds contain diagnostic fragments of sulfonated molecules (*m*/*z* 96.96 HSO_4_^−^) and multiple fragments resulting from sequential losses of chlorines as HCl ([M − 36]^−^). Molecular networking groups these metabolites into a large molecular family of structurally related compounds (Supplementary Figure S10) highlighting the potential for undescribed chemical diversity in this compound class.

**Figure 5:**
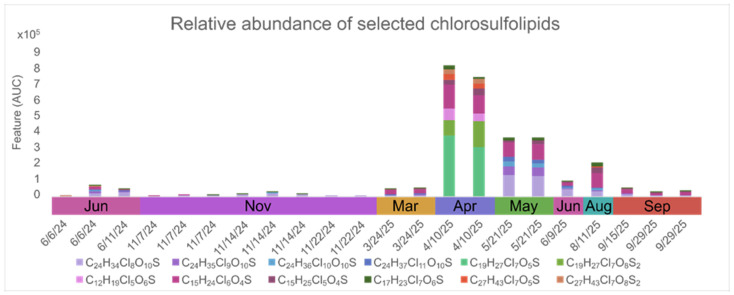
Relative abundance of the most common chlorosulfolipids detected in the Scripps Pier extracts. The highest levels coincide with algal blooms (mostly *Pseudo-nitzschia* and *Alexandrium catenella*) in April and May 2025 that followed an upwelling event on 3 April 2025.

The predicted molecular formulas for twelve chlorosulfolipids allowed us to group them into six classes based on carbon chain length: C_12_, C_15_, C_24_, and the unprecedented chain lengths C_17_, C_19_, and C_27_, with the latter bearing up to two sulfate groups. The highest chlorosulfolipid levels were found in April and May 2025 and coincided with multiple *Pseudo-nitzschia* spp. and *Alexandrium catenella* blooms that followed an upwelling event in early April 2025 (Figure 5). Interestingly, the C_24_ chlorosulfolipids were present at the highest levels primarily in May 2025 while the C_17_, C_19_, and C_27_ chlorosulfolipids were abundant in April. To the best of our knowledge, this is the first report of chlorosulfolipids in Eastern Pacific coastal marine ecosystems. While domoic acid (DA) was assumed to be the main cause of marine mammal strandings reported in conjunction with the April–May *Pseudo-nitzschia* and *Alexandrium catenella* blooms (NOAA news report https://www.fisheries.noaa.gov/feature-story/stranding-team-responds-more-dozen-dead-or-dying-san-diego-dolphins-single-day (accessed on 2 April 2026) and California HAB Bulletin, March–April 2025 https://sccoos.org/california-hab-bulletin/march-april-2025, accessed on 20 May 2026), it was not detected in our analyses.

### 2.5. Correlation of Metabolomes with Phytoplankton Counts

**Figure 6:**
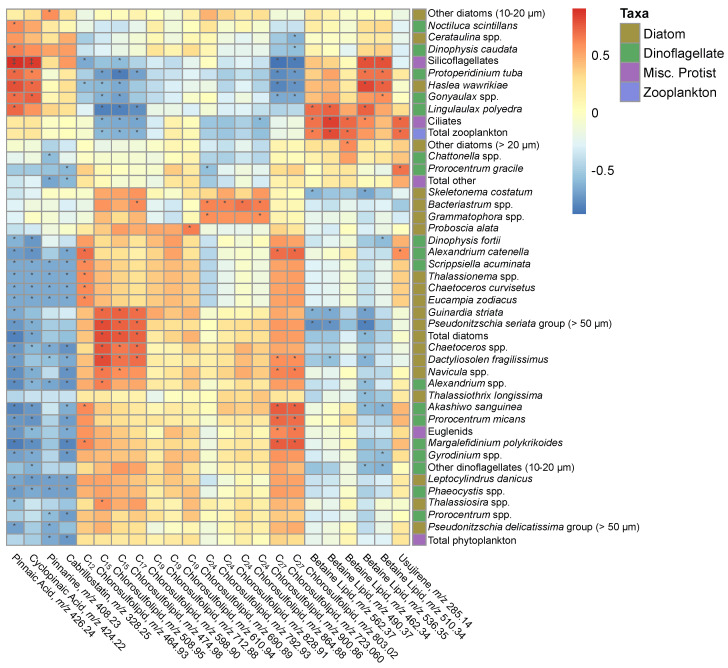
Heatmap of taxa with significant Spearman correlations to compounds identified in the SP metabolomes. Taxa (*y*-axis) with significant correlations with a compound (*x*-axis) are shown with an asterisk (*). Significant correlations above 0.85 are shown with a double dagger (‡). Warmer colors show positive correlations and cooler colors show negative correlations. Colored bars on the right indicate broad taxonomic classification.

Having identified compounds of interest in the SP deployments, we sought to identify candidate producers by correlating compound abundance with phytoplankton counts (Figure 6). In total, we assessed 27 different groups of diatoms and 19 different groups of dinoflagellates in weekly seawater samples collected from Scripps Pier. Pinnaic acid and its analogs had significant (adjusted *p* < 0.05) positive correlations with silicoflagellates (r_s_(20) > 0.86). These photosynthetic protists are associated with harmful algae blooms (HABs) and are believed to cause fish mortality by physical damage [39]. To the best of our knowledge, no natural products have been reported from this group.

Significant correlations between chlorosulfolipid abundance and phytoplankton counts varied by compound chain length. The short chain chlorosulfolipids (C_15_, C_17_) were positively correlated with several diatoms (*Pseudo-nitzschia seriata* group, *Dactyliosolen fragilissimus*, *Chaetoceros* spp., and *Guinardia striata*) (r_s_(20) > 0.71) while the shortest (C_12_) chlorosulfolipids were correlated (r_s_(20) = 0.69) with the dinoflagellate *Alexandrium catenella*. The longer chain (C_27_) chlorosulfolipids correlated with several dinoflagellates (*Akashiwo sanguinea*, *Prorocentrum micans*, *Margalefidinium polykrikoides;* r_s_(20) > 0.72). In contrast, the C_24_ chlorosulfolipids were correlated with the diatom *Bacteriastrum* spp. (r_s_(20) > 0.62) and to a lesser extent *Grammatophora* spp. (r_s_(20) > 0.57). These analyses suggest that different phytoplankton may produce chlorosulfolipids with different chain lengths. Chrysophytes, which have been shown to produce chlorosulfolipids in fresh water environments [34,35], were not assessed. Betaine lipids were positively correlated (r_s_(20) > 0.71) with ciliates, silicoflagellates, *Haslea wawrickae*, and *Lingulaulax polyedra* (formerly *Lingulodinium polyedra*). Finally, usujirene showed significant positive correlations (r_s_(20) > 0.68) with ciliates and the dinoflagellate *Prorocentrum gracile*. We queried cabrillostatin but did not find any significant positive correlations, suggesting that it might be produced by an organism that is not included in the phytoplankton counts.

Finally, we sought correlations between phytoplankton counts and metabolomic features that could not be identified by LCMS analysis (Supplementary Figure S11). To minimize spurious correlations, we used a stringent Spearman correlation cut-off of r_s_(20) > 0.85 and only looked at those correlations that were significant (adjusted *p* < 0.05). This analysis found a total of 81 metabolomic features across both positive and negative modes that were highly correlated with 22 phytoplankton taxa. Hierarchical clustering revealed groups of features significantly correlated with different taxa. One large group of features was correlated with a group of organisms dominated by dinoflagellates (4/6 taxa) while another group mostly correlated with diatoms (5/9 taxa, Supplementary Figure S11). Hypotheses generated from these analyses provide future opportunities to establish formal linkages if the candidate producers can be obtained in culture.

## 3. Discussion

Most of the world’s oceans’ natural product chemical space remains undescribed. Environmental metabolomics provides new opportunities to capture this chemical space, describe its composition, and understand how it varies over time and space. Here, we provide new evidence that Small Molecule In Situ Resin Capture (SMIRC) is a robust platform to access marine chemical space. Our findings reveal a high degree of chemical novelty, as evidenced by the limited number of matches to the Dictionary of Natural Products and low GNPS annotation rates. The plethora of unknown halogenated compounds detected in a single Scripps Pier metabolome supports the discovery potential offered by this technique. Given the assumption that most of these compounds are of microbial origin, it provides a culture-independent approach to access a component of natural product diversity that is largely missed using more traditional approaches.

The metabolomes captured at three sites around San Diego show evidence of spatial and temporal variation. This indicates that opportunities for discovery far exceed the compounds captured at any one place and time. Annotations based on careful MS/MS analyses allowed for the putative identification of several known compounds from the Scripps Pier metabolomes. These included members of the rare pinnaic acid class, which was originally reported from filter-feeding marine invertebrates yet are likely of microbial origin. Chlorosulfolipids were detected for the first time from the Eastern Pacific Ocean and included a compound with 11 chlorine atoms, which appears to match the highest number of chlorines reported for a natural product. The vast majority of the compounds detected could not be annotated, providing evidence that extensive chemical novelty was captured.

Given the assumption that some of the compounds originate from eukaryotic microalgae, we attempted to link compounds to their producers by correlating weekly phytoplankton counts with compound abundances. While multiple taxa were generally correlated with the same compound, all four C_24_ chlorosulfolipids were positively correlated with diatoms in the genus *Bacteriastratum*. In contrast, chlorosulfolipids of other chain lengths were almost entirely correlated with other taxa. This raises the possibility that different taxa produce chlorosulfolipids of different chain lengths. Yet linking captured metabolites to their producers using this approach remains challenging. Given that our comparisons were limited to specific phytoplankton taxa, other biological sources could have been missed. Furthermore, the relationships between compound persistence in the environment and producer lifespan remain unknown. Another consideration is that resins were deployed for up to four days (Scripps Pier) while phytoplanktonic community composition was measured at a single time point and can vary on a scale of hours to days [40]. Finally, covarying taxa make it challenging to discriminate among producers. Despite these challenges, correlation analyses can provide a first step towards identifying candidate natural product producers.

Regarding refinements to the SMIRC method, we found that HP-20 resin can be reused following extraction and re-activation, albeit with lower overall extract yields. We compared ACS- and HPLC-grade methanol for washing and activating the HP-20 resin before deployment and found little difference in terms of solvent-derived impurities. However, we found that using recycled methanol resulted in unacceptable levels of PEG polymer contamination in the extracts. Overall, the cost and effort associated with capturing compounds directly from the environment are small compared to screening extracts derived from microbial libraries.

While the field of environmental metabolomics continues to develop, it has become clear that direct resin capture provides new opportunities for natural product discovery. Bioassays can readily be incorporated into the workflow to guide the isolation of biologically active compounds while mass spectrum features, such as isotopic patterns, can be used to target compounds of interest. While further research is needed to strengthen producer–compound linkages, direct capture methods provide unique opportunities to access unexplored chemical space.

## 4. Materials and Methods

### 4.1. Resin Preparation

The polystyrene/divinylbenzene matrix Diaion^TM^ HP-20 resin (Thermo Fisher Scientific Inc., Waltham, MA, USA) was soaked in ACS-grade methanol (MeOH) for 30 min and then filtered through a glass fritted Büchner funnel under vacuum. In the funnel, the resin was washed 3× times with ACS-grade MeOH, 2× times with DI water, and once with high-purity MilliQ water. The solvent volumes used for washing were sufficient to fully submerse the resin. Up to 200 g of moist resin was put inside a folded sheet of nylon mesh (Nitex™, pore size 120 µm, Genesee Scientific, El Cajon, CA, USA) and tightly enclosed inside a 30 cm wooden embroidery hoop. In some cases, the resin was prepared in advance and stored for several days in MilliQ water at room temperature before deployment. The agar-resin matrix consisted of 500 g activated HP-20 resin, 1 L artificial seawater (26 g/L of Instant Ocean in DI water), and 10 g agar, which was autoclaved at 121 °C for 30 min. After cooling to 50 °C, the liquid mixture was swirled and poured into ten 15 cm plastic Petri dishes. Two solidified agar/resin plates were aseptically removed from the dishes and enclosed between two layers of Nitex™ nylon mesh as described above. The Nitex™ mesh and embroidery hoops were treated with 70% isopropanol and allowed to dry before packaging. Packaged containers were wrapped with 70% isopropanol-treated aluminum foil prior to deployment.

### 4.2. Deployment and Extraction

The packaged resin containers were placed on the sea floor (SCUBA or snorkeling) and secured using aluminum tent stakes or rocks (MB and MR deployments) or attached with nylon ropes inside the sea water supply channel on Scripps Pier. Deployment duration was from 20 min to 14 days. Upon recovery, individual resin packages were washed with deionized water and the resin removed from the nylon mesh and transferred into a glass fritted Büchner funnel (200 µm pore size) where it was rinsed with MilliQ H_2_O to remove residual seawater. The resin was then eluted three times by adding 150 mL HPLC-grade MeOH per 100 g HP-20, allowing it to soak for 20 min, and then recovering the MeOH using vacuum. The extracts were combined and the solvent removed on a rotary evaporator to yield a salt-free crude extract. Soft agar-resin plates were crushed and processed in the same way.

### 4.3. LCMS Analysis

LC-HRMS/MS was performed on an Agilent 6538 Accurate-Mass QToF with ESI-source coupled with an Agilent 1290 Infinity UHPLC system (Agilent Technologies, Santa Clara, CA, USA) equipped with a degasser, binary pump, autosampler, column oven (30 °C), DAD detector, and a Luna Omega 1.6 µm, Polar C_18_ 100 × 2.1 mm column (Phenomenex, Torrance, CA, USA). The mass spectrometer was calibrated using the Agilent Reference Calibration Mix. Crude extracts (5 µL, 5 mg/mL) were injected into the LC-HRMS and eluted at 250 µL/min, using a mobile phase gradient (A: acetonitrile with 0.1% formic acid and B: H_2_O with 0.1% formic acid) starting with 20% A to 60% A over 10 min, then increasing to 100% A over 5 min and finishing with 100% A for 2 min before equilibrating back to 20% A over 2 min. For the analysis of chlorosulfolipids, the same system was used with a Kinetex 2.6 µm, Polar C_18_ 50 × 2.1 mm column (Phenomenex, Torrance, CA, USA). The LC program was isocratic at 400 µL/min with 10% A for 1 min and then gradually increased to a gradient to 100% A over 12 min and finished with 100% A for 2 min before equilibrating back to 10% A. MS1 data were acquired over the range 155–1700 *m*/*z* and MS2 over 80–1700 *m*/*z* in positive and negative modes using the following parameters: MS scan rate, 2/s; MS/MS scan rate, 4/s; gradient collision energy (slope 2.6, offset 15 eV); source gas temperature, 300 °C; drying gas flow rate 5 L/min. All solvents were LCMS-grade. Manual data analysis was performed using the Masshunter Qualitative Analysis Software B.05.00 (Agilent Technologies, Santa Clara, CA, USA).

### 4.4. Mass Spectrometry Data Analysis

Agilent .d files were converted to centroided .mzXML format using the GNPS Vendor Converter. Metabolome feature lists were generated with MZmine v4.8.5 [41]. Mzwizard was used to set the parameters to HPLC QToF and data-dependent analysis (DDA). MS1 and MS2 mass detection was performed at noise level of 5E3 and 1E2, respectively. For the chromatogram builder, the minimum number of consecutive scans was set to 3 with a minimal intensity of 6E3 and *m*/*z* tolerance of 0.005 or 20 ppm. Chromatogram deconvolution was achieved with the Local Minimum Feature Resolver using a chromatographic threshold of 71.2%, a minimum search range of 0.15 min, a minimum absolute height of 6E3 and a minimum ratio of peak top/edge of 1.4. Minimum consecutive scans were set to 3 and peak duration range was set to 0–4.5 min. MS1 to MS2 precursor tolerance was set to 20 ppm and a minimum relative feature height to 0.25. Features without an MS/MS spectrum were filtered from the lists. Join Aigner was used to merge the feature lists with an *m*/*z* tolerance of 8 ppm and a retention time tolerance of 0.15 min. Feature lists were gap filled with an intensity tolerance of 0.2, *m*/*z* tolerance of 0.005 or 20 ppm, and a retention time tolerance of 0.2 min. Feature tables, MS/MS spectra, and Ion Identity Molecular Networking (IIMN) edges [42] were exported to the Feature-Based Molecular Networking [43] workflow (FBMN). Molecular networks were created using GNPS2 Analysis Hub (closed beta testing) and the Global Natural Products Social Networking (GNPS) environment [10] using the following settings: MS/MS fragment ions within +/− 17 Da of the precursor *m*/*z* were removed, MS/MS spectra were window filtered by choosing only the top six fragment ions in the +/− 50 Da window throughout the spectrum. The precursor ion mass tolerance was set to 0.05 Da with an MS/MS fragment ion tolerance of 0.05 Da. Edges were filtered to a cosine score above 0.65 and more than four matched peaks. Edges were kept only if each of the nodes appeared in each other’s respective top 10 most similar nodes. The maximum size of a molecular family was set to 100 with the lowest scoring edges removed until the molecular family size was below this threshold. All GNPS annotations were required to have a cosine score above 0.7 and at least 4 matched peaks. Molecular networks were visualized in Cytoscape version 3.8 [44]. FBMN-STATS [45] was used to generate blank-removed feature tables with a blank removal threshold of 0.15. Molecular formulas were predicted with SIRIUS v6.3.3 [46] and the ChemCalc online tool version 3.4.1 https://www.chemcalc.org/ (accessed on 2 March 2026) [47] under consideration of ^12^C to ^13^C isotopic peak ratio (=approximate number of carbons) and chromatographic retention time for plausible level of oxygenation and isotopic patterns for halogenated molecules. The Dictionary of Natural Products (DNP) database was queried with predicted molecular formulas and accurate masses (after ppm error correction). The GNPS jobs can be accessed under https://gnps2.org/status?task=407343d99c9e4b5fbf297cf830f2503a (positive mode, accessed on 2 July 2026) and https://gnps2.org/status?task=1a7985c3ac2d4d43b12865925e6b618c (negative mode, accessed on 2 July 2026).

### 4.5. Phytoplankton Cell Counts

Eukaryotic phytoplankton were enumerated weekly from the Scripps Pier node of the SCCOOS HABMAP (http://sccoos.org/harmful-algal-bloom, accessed on 2 July 2026) and the McGowan Plankton Chlorophyll Program, as previously described [48,49]. Diatom and dinoflagellate abundances were enumerated by settling 10–50 mL of seawater preserved with 4% formaldehyde and then identifying and counting cells through a phase-contrast, inverted light microscope at 200× amplification to the lowest taxonomic group (species or genus) feasible. To maintain consistency in the groups of plankton counted, some species were combined into genus-level groups. These include total counts for diatoms and dinoflagellates, and four subgroups for the genus *Pseudo-nitzschia* based on cell length and valve width (with *Pseudo-nitzschia delicatissima* group with valve width less than 3 µm and cell length less than or greater than 50 µm, *Pseudo-nitzschia seriata* group with valve width greater than 3 µm and cell length less than or greater than 50 µm).

### 4.6. Correlation Analysis

Positive- and negative-mode MS feature tables and phytoplankton count tables were imported into R (4.5.1) and RStudio (2026.01.0, Build 392). The packages “tibble” and “dplyr” (1.2.0) were used to clean and prepare the data tables for downstream analysis. The metabolomic data was preprocessed in R to impute small values (lowest intensity divided by 2) in replacement of any 0 s and then normalized by total ion count to generate proportional data. Data for Spearman’s coefficient analysis was scaled and centered using the base R function scale. Taxa counts were log transformed via the log1p function and scaled as per the metabolomics data using the scale function. The “vegan” (2.7-2) package in R was used to generate Bray–Curtis distance matrices on unscaled metabolomics data and to generate NMDS plots, which were visualized via the “ggplot2” (4.0.2) package. Spearman coefficients were calculated using the rcorr function from the “Hmisc” (5.2-5) package and *p*-values were adjusted using the p.adjust function from the “stats” (4.5.1) package using the Benjamini–Hochberg method. Heatmaps were generated using the pheatmap function from the “pheatmap” (1.0.13) package.

## Figures and Tables

**Table 1:** Halogenated compounds detected by HR-MS (positive mode) from a single Scripps Pier extract (SP24-R2). DNP: Dictionary of Natural Products. Note: slightly different *m*/*z* values for pinnaic acid analogs are reported in Supplemental Figures S6–S8, which were acquired using MS/MS.

*m*/*z*	Ion	RT Min	Halogen	Molecular Formula (Method)	DNP-Hits	Compound
359.1311	M + H	3.41	Br	C_16_H_27_BrN_2_O_2_ (SIRIUS)	0	
583.2658	M + H	3.69	Cl	C_29_H_43_ClN_2_O_8_ (chemcalc)	0	
424.2217	M + H	4.49	Cl	C_23_H_34_ClNO_4_ (SIRIUS)	0	
426.2375	M + H	5.18	Cl	C_23_H_36_ClNO_4_ (SIRIUS)	1	Pinnaic acid
281.0626	M + H	5.22	Br	C_10_H_19_BrNO_3_ (SIRIUS)	0	
449.1585	M + H	5.73	Cl_2_	C_20_H_30_Cl_2_N_2_O_5_ (chemcalc)	0	
405.1734	M + H	5.87	Cl_2_	C_19_H_30_Cl_2_N_2_O_3_ (chemcalc)	0	
408.2266	M + H	6.15	Cl	C_23_H_34_ClNO_3_ (SIRIUS)	1	Pinnarine
592.2024	M + H	6.83	Cl_2_	C_27_H_39_Cl_2_NO_9_ (chemcalc)	0	
445.1988	M + H	7.16	Cl_2_	C_22_H_34_Cl_2_N_2_O_3_ (chemcalc)	0	
574.1923	M + H	7.44	Cl_2_	C_27_H_37_Cl_2_NO_8_ (chemcalc)	0	
588.2075	M + H	7.99	Cl_2_	C_28_H_39_Cl_2_NO_8_ (chemcalc)	0	
864.3539	M + H	9.09	Cl_3_	C_42_H_64_Cl_3_NO_11_ (chemcalc)	0	
558.1953	M + H	10	Cl	C_28_H_32_ClN_3_O_7_ (chemcalc)	0	
618.3115	M + H	11.07	Cl	C_29_H_48_ClN_3_O_9_ (chemcalc)	0	
644.3866	M + H	11.23	Cl	C_34_H_58_ClNO_8_ (chemcalc)	0	
724.352	M + H	11.23	Cl	C_36_H_54_ClN_3_O_10_ (chemcalc)	0	
602.3194	M + H	11.68	Cl	C_34_H_48_ClNO_6_ (chemcalc)	0	
586.3244	M + H	13.87	Cl	C_34_H_48_ClNO_5_ (chemcalc)	0	

## Data Availability

Mass spectrometry data was deposited and made publicly available at massive.ucsd.edu under MSV000102013.

## Supplementary Materials

The following supporting information can be downloaded at: https://www.mdpi.com/article/10.3390/md24070237/s1, Table S1: SMIRC Deployments; Table S2: Top 22 GNPS MS/MS spectral matches (positive mode); Table S3: Top 18 GNPS MS/MS spectral matches (negative mode); Figure S1: High confidence spectral matches (positive mode, MS/MS mirror plots); Figure S2: High confidence spectral matches (negative mode, MS/MS mirror plots); Figure S3: Molecular network reveals a molecular family of betaine lipids; Figure S4: Relative abundance of major betaine lipids across Scripps Pier extracts; Figure S5: Molecular network showing malyngamide molecular family detected in Mission Bay extracts; Figure S6: Base Peak Chromatogram of the SP24-R2 crude extract and manually annotated MS/MS spectrum of pinnaic acid; Figure S7: Manually annotated MS/MS spectrum of pinnarine; Figure S8: Manually annotated MS/MS spectrum of putative new compound cyclopinnaic acid; Figure S9: Relative abundance of pinnaic acid and its congeners in Scripps Pier extracts; Figure S10: Molecular network showing the extensive chlorosulfolipid molecular family; Figure S11: Heatmap of significant Spearman correlations between taxa and unidentified MS features.
