# Supplementary material for: Direct Capture Methods Reveal Extensive Organohalide Chemical Space in Marine Environments
Source: Mar Drugs. 2026 Jul 4;24(7):237. doi: 10.3390/md24070237 (PMC13413282; doi:10.3390/md24070237)
Supplement: Supplementary file 1 [file marinedrugs-24-00237-s001.zip › marinedrugs-4379009-supplementary.pdf]

Supplementary Tables and Figures

Supplementary Table S1. SMIRC deployments. NA: not applicable.

Deployment Nr-replicate	Extract ID	Date	Hours	Site	Depth m	Resin type	Resin amount g	Extract amount mg	Yield mg/g resin
1-1	SP24_R1	6/6/2024	0.33	SP	NA	HP20	100	80	0.80
1-1	SP24_R2	6/6/2024	20	SP	NA	HP20	100	114	1.14
2-1	SP24_R5	6/11/2024	20	SP	NA	used_HP20	100	102	1.02
3-1	SP24_R6	11/7/2024	24	SP	NA	HP20	130	200	1.54
3-2	SP24_R7	11/7/2024	24	SP	NA	HP20	130	174	1.34
3-3	SP24_R8	11/7/2024	96	SP	NA	HP20	130	360	2.77
4-1	SP24_R9	11/14/2024	96	SP	NA	HP20	150	300	2.00
4-2	SP24_R10	11/14/2024	96	SP	NA	HP20	150	400	2.67
4-3	SP24_R11	11/14/2024	96	SP	NA	HP20	150	440	2.93
5-1	SP24_R12	11/22/2024	96	SP	NA	HP20	150	350	2.33
5-2	SP24_R13	11/22/2024	96	SP	NA	HP20	150	581	3.87
6-1	SP25_R1	3/24/2025	48	SP	NA	HP20	125	208	1.66
6-2	SP25_R2	3/24/2025	48	SP	NA	HP20	125	140	1.12
7-1	SP25_R3	4/10/2025	24	SP	NA	HP20	130	96	0.74
7-2	SP25_R4	4/10/2025	24	SP	NA	HP20	130	150	1.15
8-1	SP25_R5	5/21/2025	48	SP	NA	HP20	100	150	1.50
8-2	SP25_R6	5/21/2025	48	SP	NA	HP20	100	159	1.59
9-1	SP25_R7-8	6/9/2025	48	SP	NA	HP20	150	280	1.87
10-1	SP25_R9	8/11/2025	48	SP	NA	HP20	150	212	1.41
11-1	SP25_38R	9/15/2025	24	SP	NA	HP20	300	414	1.38
12-1	SP25_40R1	9/29/2025	24	SP	NA	used_HP20	150	220	1.47
12-2	SP25_40R2	9/29/2025	24	SP	NA	HP20	150	245	1.63
13-1	MB24_R1	8/1/2024	160	MB	1	used_HP20	150	39	0.26
13-2	MB24_R2	8/1/2024	160	MB	1	used_HP20	150	33	0.22
13-3	MB24_R3	8/1/2024	160	MB	2	used_HP20	150	118	0.79
14-1	MR24_R5	9/9/2024	216	MR	16	HP20	100	220	2.20
14-2	MR24_AR3	9/9/2024	216	MR	16	HP20-agar	150	332	2.21
14-3	MR24_AR4	9/9/2024	216	MR	16	HP20-agar	150	400	2.67
14-4	MR24_AR5	9/9/2024	216	MR	16	HP20-agar	150	295	1.97
14-5	MR24_AR6	9/9/2024	216	MR	16	HP20-agar	150	250	1.67
15-1	MR25_R1	5/23/2025	168	MR	17	HP20	150	152	1.01
15-2	MR25_R2	5/23/2025	168	MR	17	HP20	150	104	0.69
15-3	MR25_R3	5/23/2025	168	MR	17	HP20	150	106	0.71
15-4	MR25_R4	5/23/2025	168	MR	17	HP20	150	91	0.61

Supplementary Table S2. Top 22 GNPS MS/MS spectral matches (positive mode).

ID	Cosine Score	Shared Peaks	Precursor m/z	Spectrum m/z	Mass Diff.	Compound Name	Adduct
9317	0.79	29	1036.69	1036.68	0.0088	Surfactin C1	M+H
3940	0.80	16	516.299	516.294	0.0046	Taurocholic acid	M+H
7870	0.93	12	496.34	496.336	0.0043	Lyso PC (16:0/0:0)	M+H
8217	0.85	12	522.355	522.351	0.0040	Lyso PC (18:1/0:0)	M+H
5728	0.79	12	373.274	373.271	0.0034	Cholic acid	[M+H-$2H_2O$]
6605	0.91	11	468.308	468.305	0.0030	Lyso PC (14:0/0:0)	M+H
4863	0.80	11	464.283	464.278	0.0045	Taurohyodeoxycholic acid	M-$2H_2O$+H
8304	0.71	11	327.231	327.23	0.0007	11-Hydroxy-docosahexaenoic acid	M+H-H_2O
9399	0.91	10	256.3	256.262	0.0382	Palmitamide	M+H
9191	0.81	9	311.292	311.292	0.0003	14(Z)-Eicosenoic acid	M+H
9347	0.76	9	659.431	659.425	0.0060	Fucoxanthin	M+H
7975	0.98	8	490.369	490.37	0.0009	MGCC (16:0/0:0)	M+H
7024	0.93	4	536.353	536.354	0.0036	MGCC (20:5/0:0)	M+H
9239	0.92	6	852.5464	852.554	0.0076	PC (20:5/22:6)	M+H
9238	0.94	8	778.536	778.532	0.0044	PC (18:3/18:3)	M+H
2363	0.84	8	243.088	243.086	0.0021	Lumichrome	M+H
7327	0.80	8	357.278	357.276	0.0023	3β-hydroxy-5-cholenoic acid	M+H-H_2O
7426	0.79	8	520.341	520.336	0.0046	Lyso PC (18:2/0:0)	M+H
7571	0.74	8	301.216	301.2140	0.0025	17,18-Epoxyeicosatetraenoic acid	M+H-H_2O
6061	0.76	4	328.246	328.2500	0.0043	Cabrillostatin	M+H
177	0.83	6	285.144	285.145	0.001	Usujirene	M+H

Supplementary Table S3. Top 18 GNPS MS/MS spectral matches (negative mode).

ID	Cosine Score	Shared Peaks	Precursor m/z	Spectrum m/z	Mass Diff.	Compound Name	Adduct
8322	0.86	12	714.506	714.507	0.0014	PE (16:1/18:1)	M-H
2743	0.77	11	299.056	299.057	0.0008	Diosmetin	M-H
7366	0.84	10	297.141	297.153	0.0121	Decylbenzenesulfonic acid	M-H
8224	0.76	9	599.32	599.322	0.0017	PI (18:0/0:0)	M-H
7893	0.76	9	311.15	311.169	0.0189	Pyrenophorol	M-H
9062	0.82	8	686.477	686.478	0.0006	PE (16:1/16:1)	M-H
8468	0.82	8	714.506	714.508	0.0016	PE (16:1/18:1)	M-H
7262	0.72	8	571.294	571.289	0.0045	PI (16:0/0:0)	M-H
6649	0.79	6	450.262	450.262	0.0003	PE (16:1/0:0)	M-H
8067	0.72	6	688.492	688.492	0.0002	PE (16:0/16:1)	M-H
7944	0.70	6	566.345	566.346	0.0010	Lyso PC (18:1/0:0)	M+HCOO
7699	0.70	6	311.15	311.169	0.0188	Pyrenophorol	M-H
785	0.89	5	165.056	165.056	0.0002	3-(3-Hydroxyphenyl)-propionic acid	M-H
8021	0.83	5	483.272	483.273	0.0010	PG (16:0/0:0)	M-H
1553	0.73	5	359.077	359.078	0.0014	Rosmarinic acid	M-H
8066	0.71	5	483.272	483.273	0.0010	PG (16:0/0:0)	M-H
7377	0.76	4	452.278	452.278	0.0001	PE (16:0/0:0)	M-H
5186	0.71	4	471.242	471.207	0.0353	Sulfoursodeoxycholic acid (SUDCA)	M-H

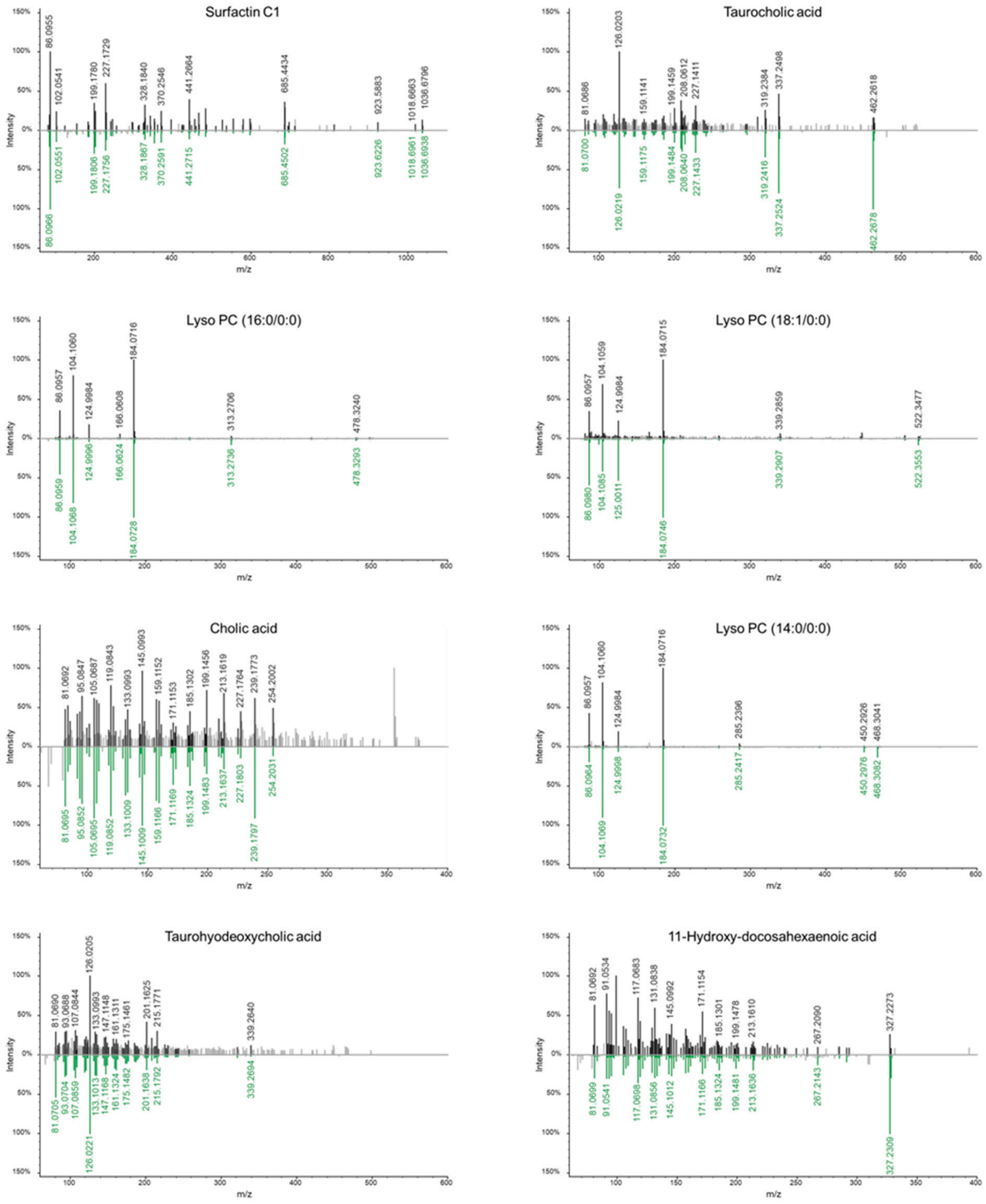

Supplementary Figure S1. High confidence spectral matches (positive mode, MS/MS mirror plots). Top (black) experimental spectrum, bottom (green) GNPS library spectrum.

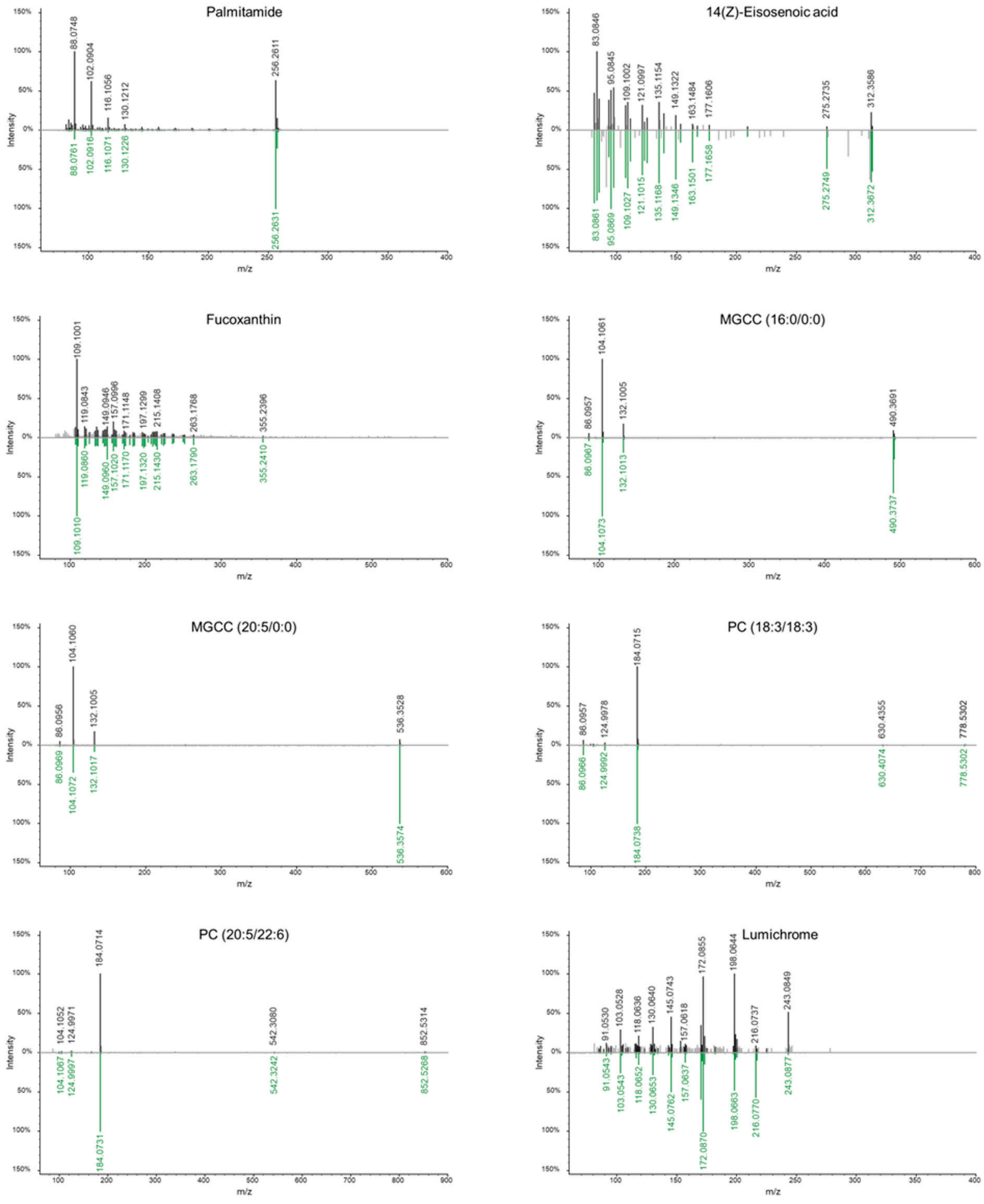

Supplementary Figure S1. Continued.

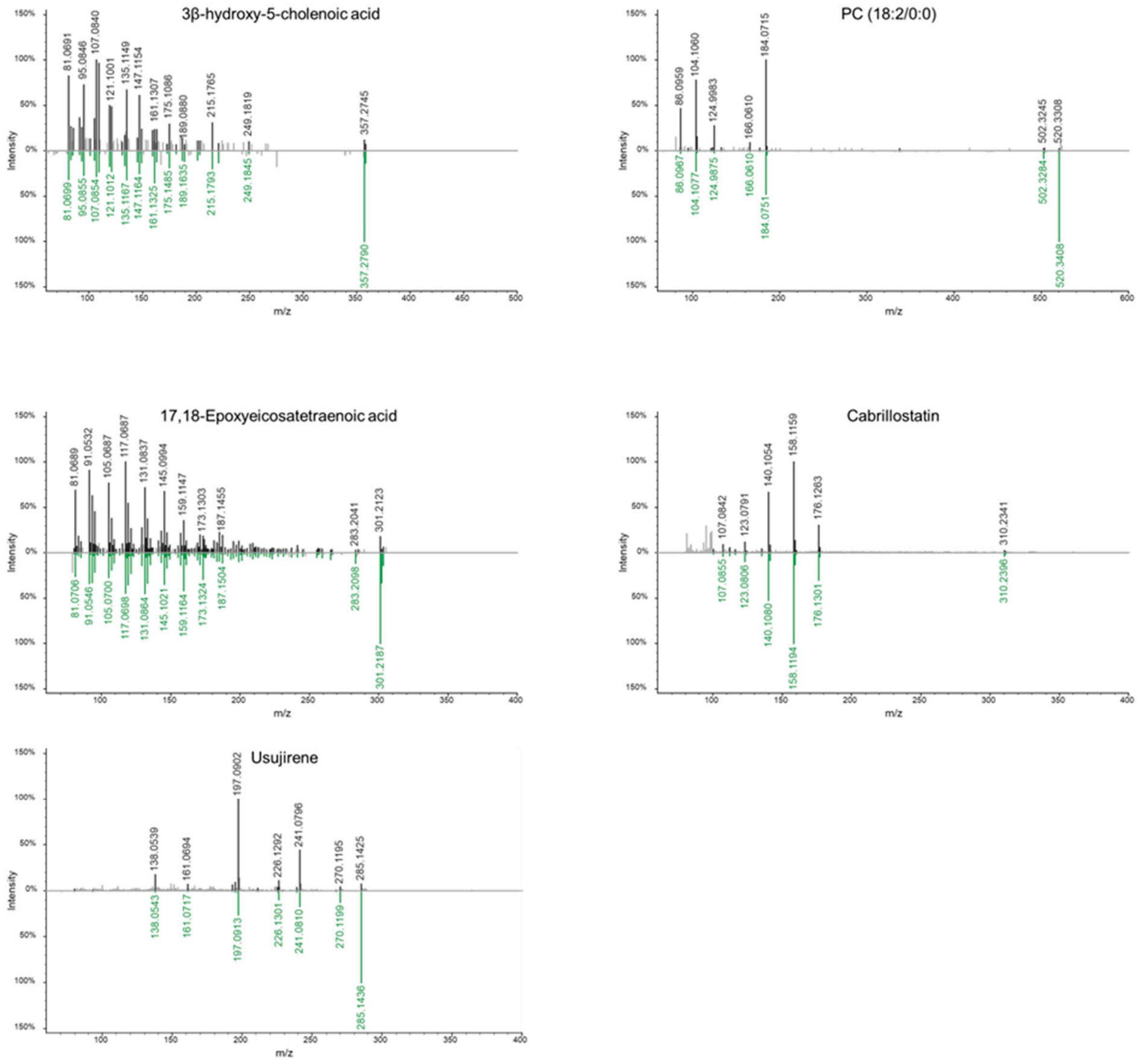

Supplementary Figure S1. Continued.

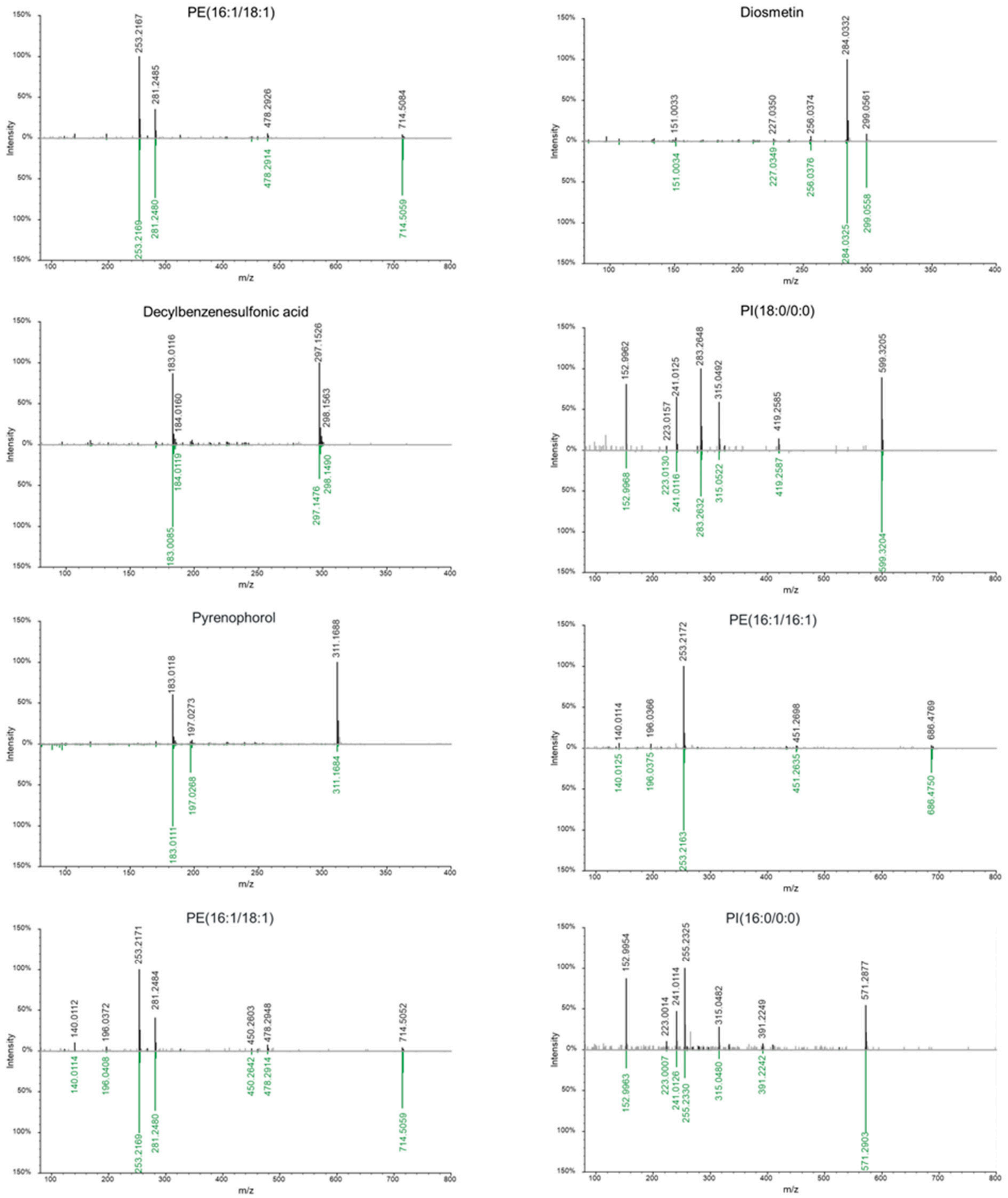

Supplementary Figure S2. High confidence spectral matches (negative mode, MS/MS mirror plots). Top (black) experimental spectrum, bottom (green) GNPS library spectrum.

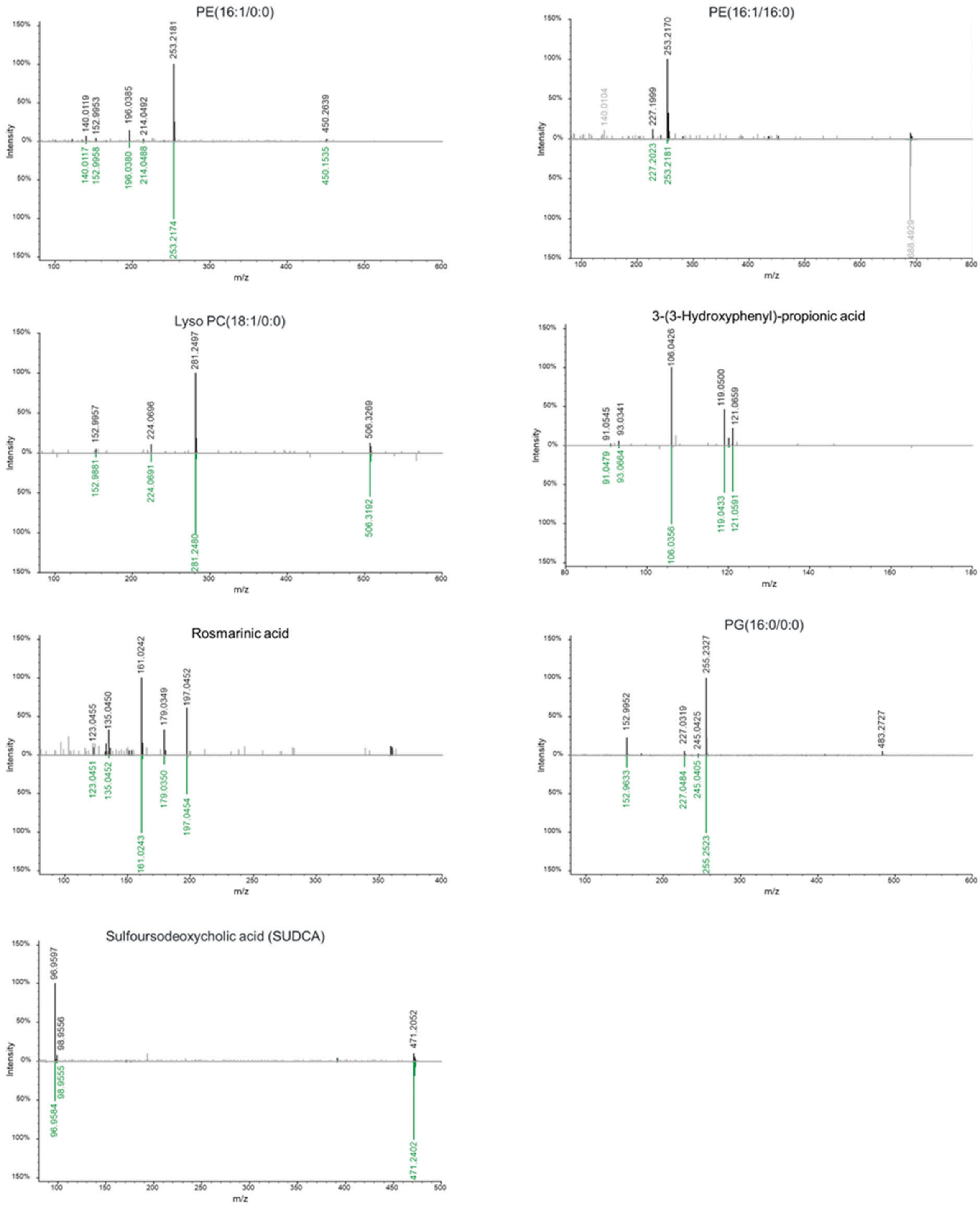

Supplementary Figure S2. Continued.

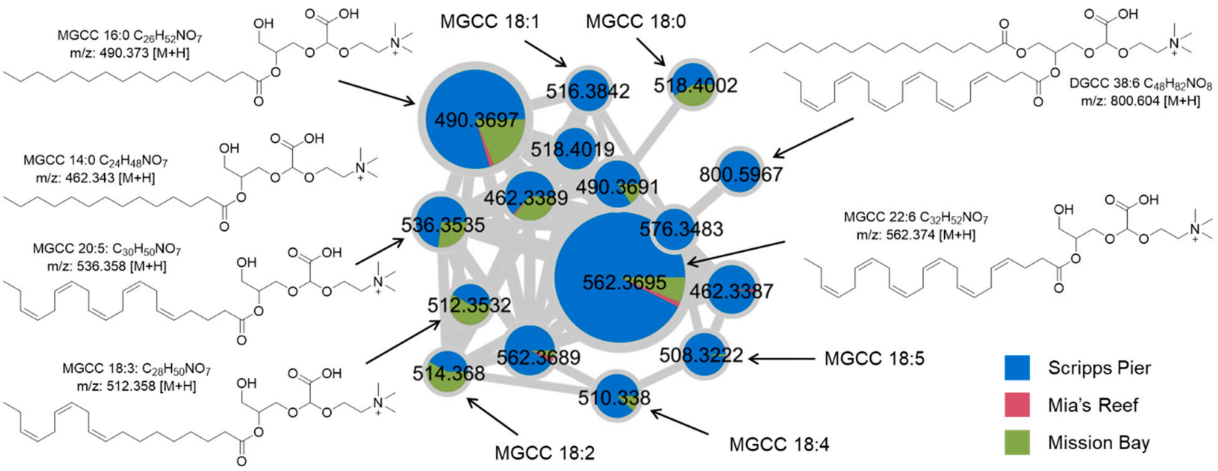

Supplementary Figure S3. Molecular network reveals a molecular family of betaine lipids. Nodes are displayed as pie charts colored by site. Node size scales with AUC for each feature. The largest nodes are linked to MGCC 16:0 *m/z* 490.373 and MGCC 22:6 *m/z* 562.374.

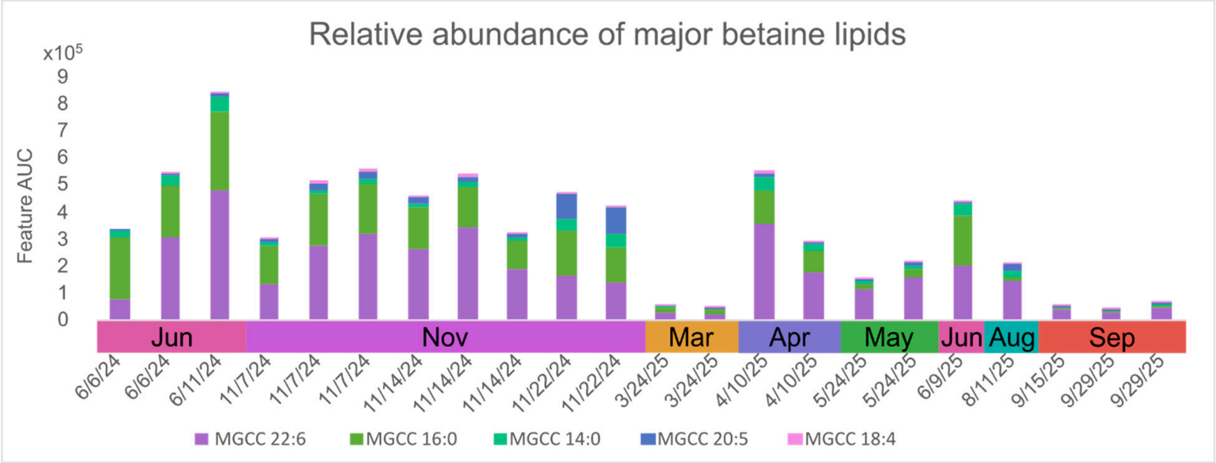

Supplementary Figure S4. Relative abundance of major betaine lipids across Scripps Pier extracts. Elevated levels were observed in June 2024 (*Akashiwo* bloom) and throughout November 2024 (*Lingulaulax* bloom).

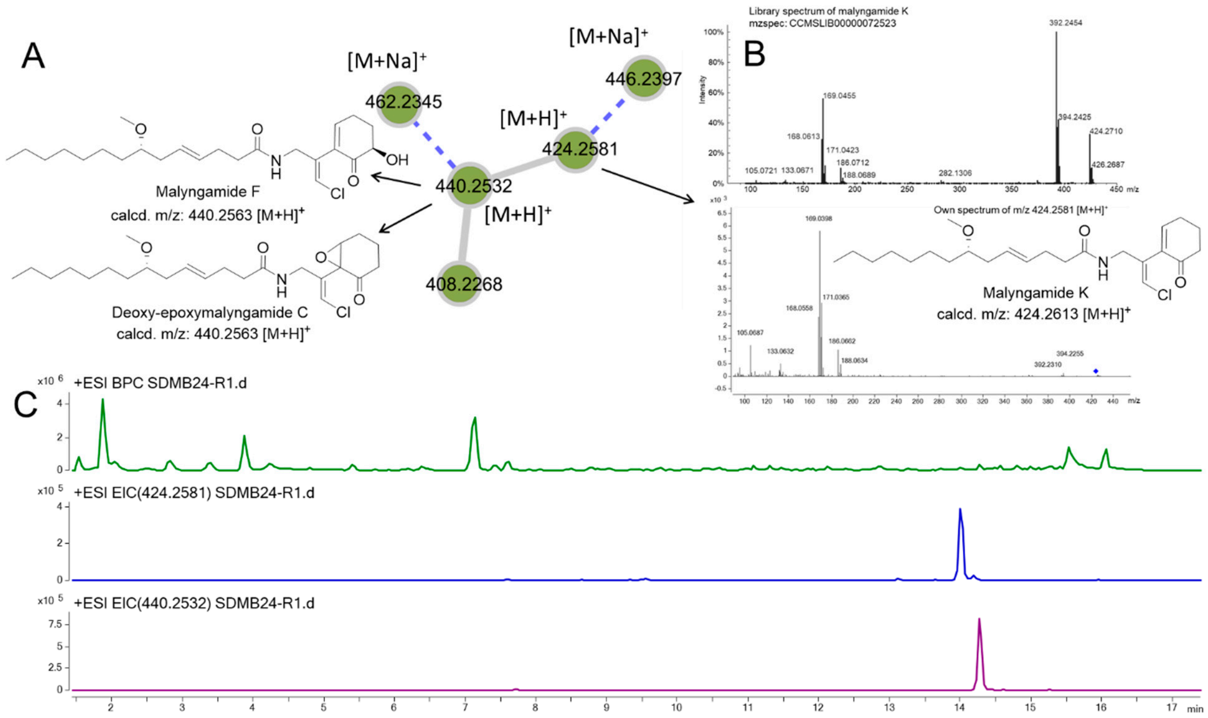

Supplementary Figure S5. A. Molecular network showing malyngamide molecular family detected in Mission Bay extracts. Dashed blue edges represents ion identity connections between different adduct ions of the same molecule. B. Top right: MS/MS library spectrum of malyngamide K. Bottom right: MS/MS spectrum from compound annotated as malyngamide K. C. BPC chromatogram of the Mission Bay extract and Extracted Ion Chromatograms (EIC) of the proposed malyngamides.

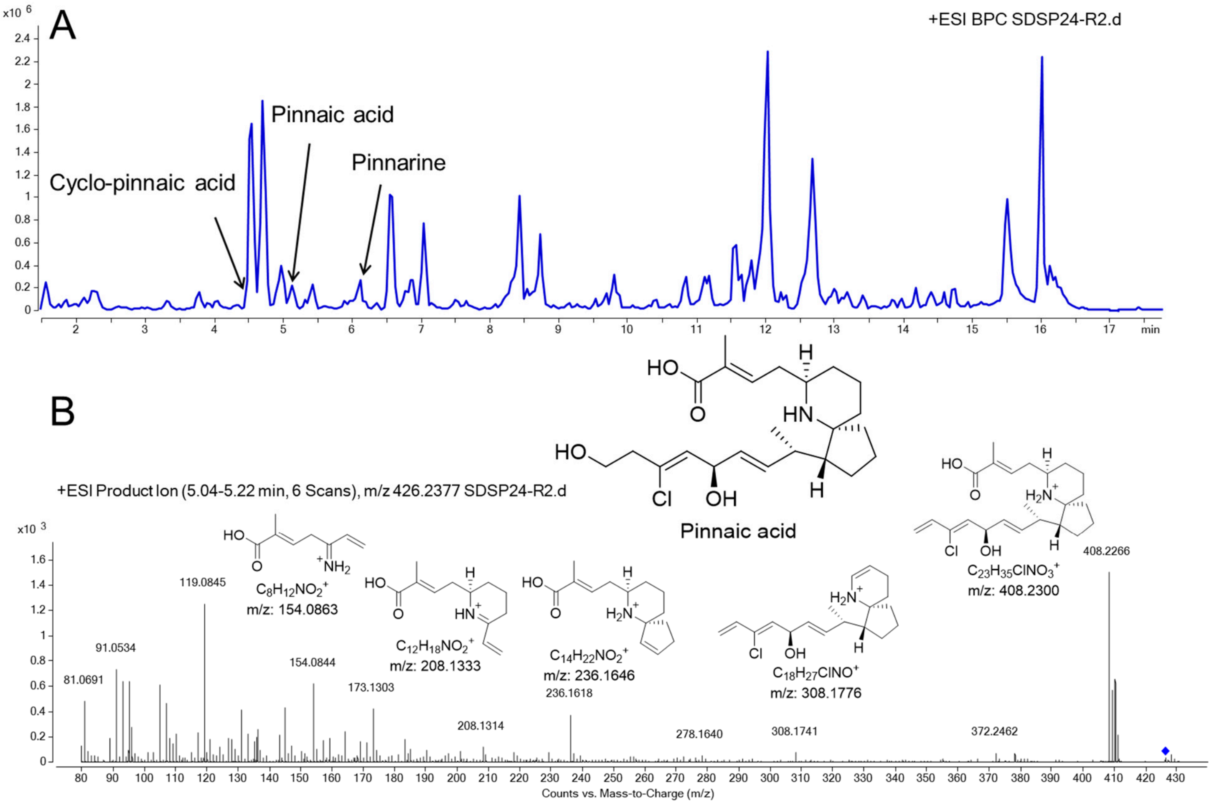

Supplementary Figure S6. A. Base Peak Chromatogram (BPC) of the SP24-R2 crude extract. Pinnaic acid and analogs are indicated with arrows. B. Manually annotated MS/MS spectrum of pinnaic acid.

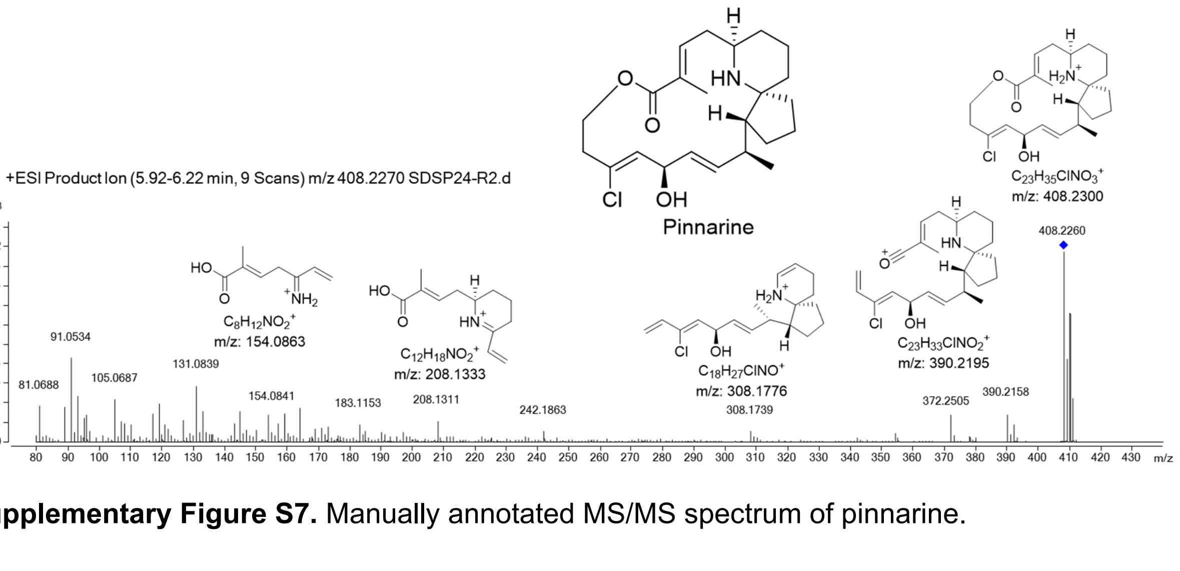

Supplementary Figure S7. Manually annotated MS/MS spectrum of pinnarine.

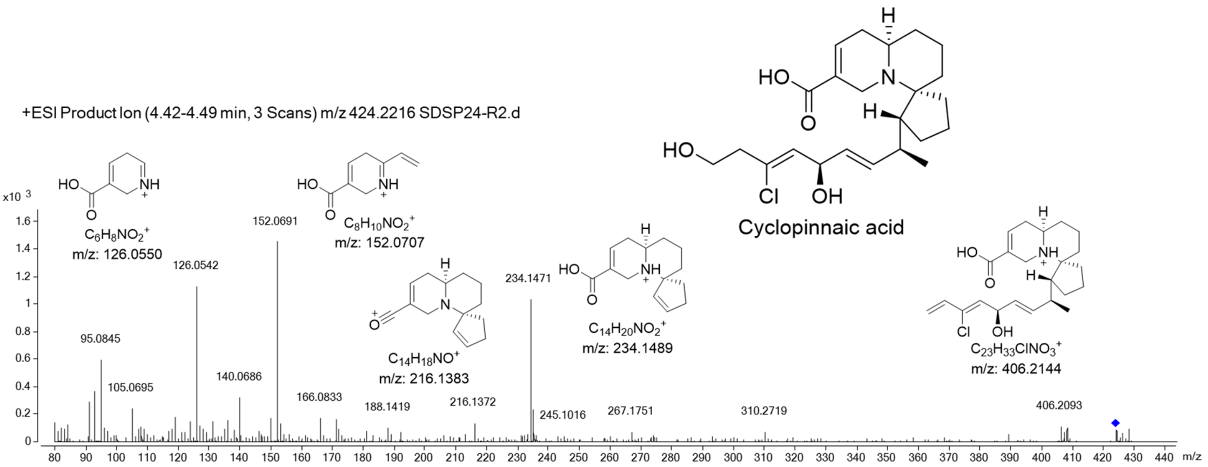

Supplementary Figure S8. Manually annotated MS/MS spectrum of putative new compound cyclopinnaic acid.

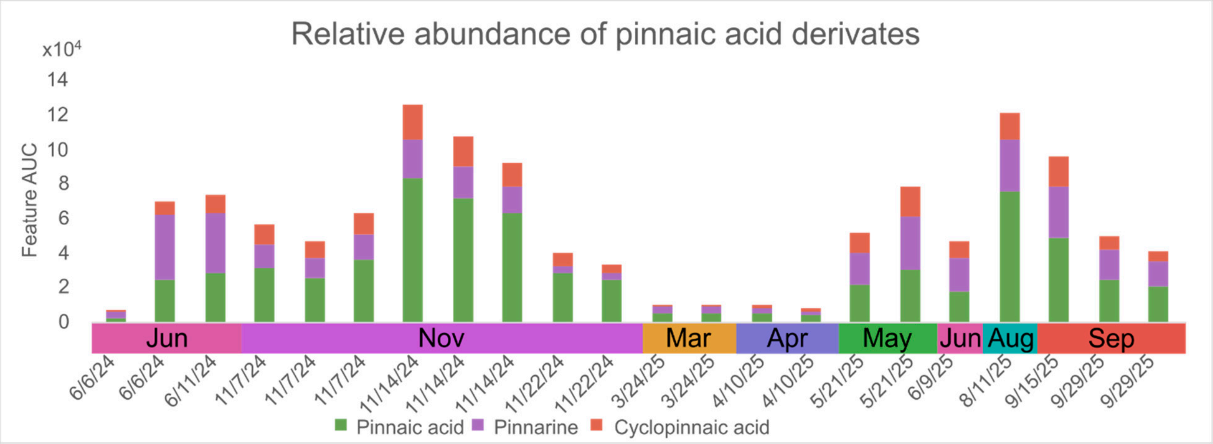

Supplementary Figure S9. Relative abundance of pinnaic acid and its congeners in Scripps Pier extracts. Booms occurred in June (*Akashiwo*) and November (*Lingulaulax*) 2024, and in March/April (*Pseudo-nitzschia* and *Alexandrium*) and June (*Akashiwo*) 2025.

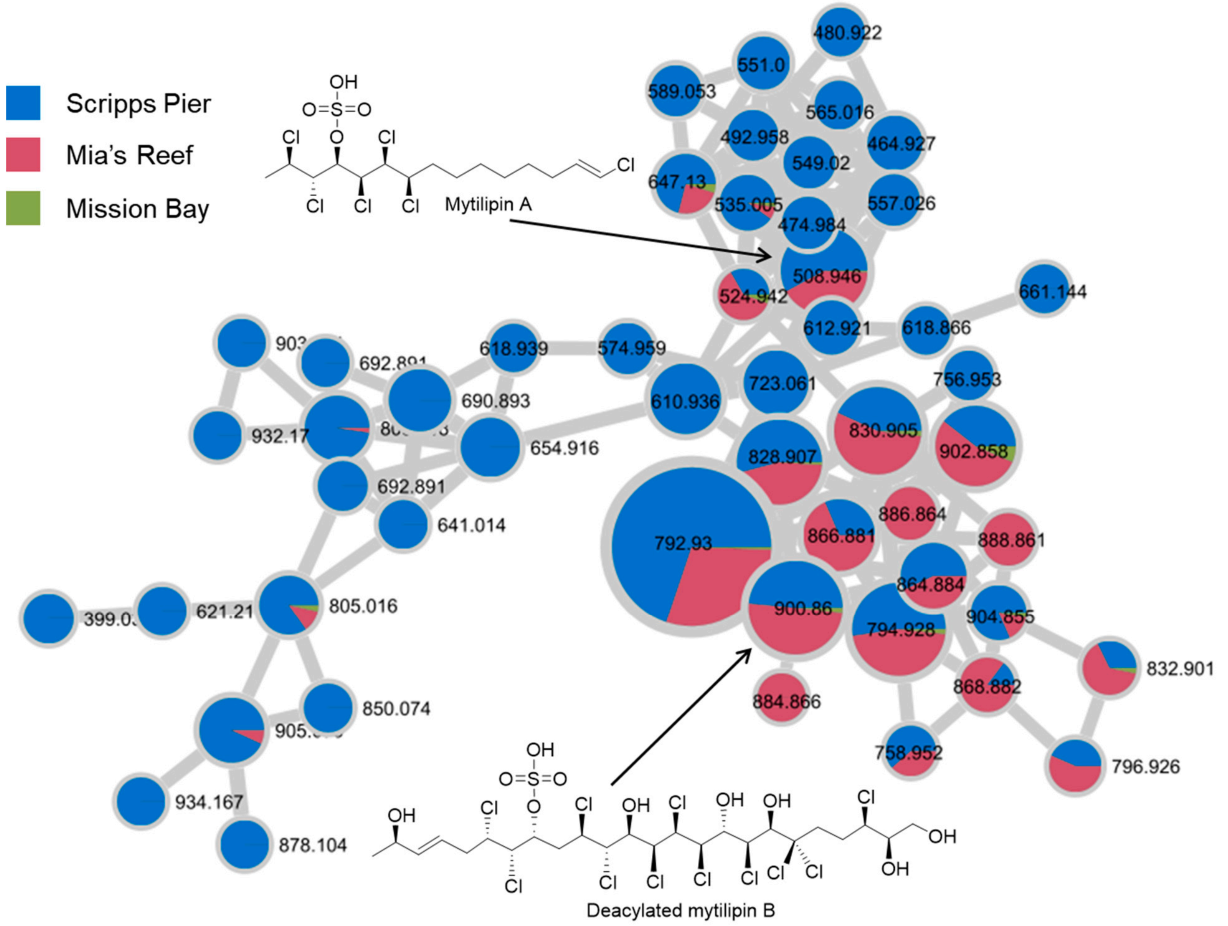

Supplementary Figure S10. Molecular network showing the extensive chlorosulfolipid molecular family. Structures putatively assigned based on MS/MS analysis. The deacylated mytilipin B is proposed as a new natural product. The stereochemistry is assigned based on literature reports for related chlorosulfolipids. Nodes are colored according to SMIRC deployment site. Node size scales with AUC for each feature.

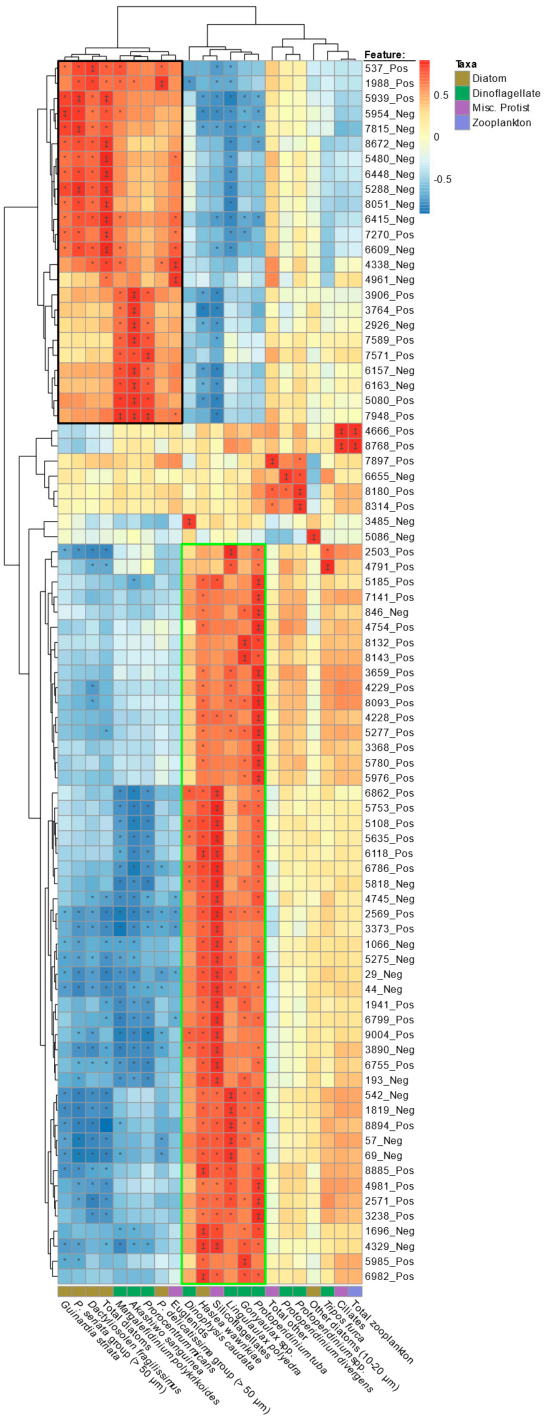

Supplementary Figure S11. Heatmap of significant Spearman correlations between taxa and unidentified MS features. Taxa (x-axis) with significant correlations to MS features (y-axis) are shown with an asterisk (*). Significant correlations above 0.85 are shown with a double dagger (‡). Warmer colors show positive correlations; cooler colors show negative correlations. Colored bars on the bottom indicates taxonomic classification. Boxes indicate compounds correlated mostly with diatoms (black) and dinoflagellates (green).
